# Relating Model Performance to Embedding Distributions in Molecular Machine Learning

**DOI:** 10.1021/acs.jcim.6c00218

**Published:** 2026-04-22

**Authors:** Matthias Welsch, Ellena Jiang, Ioannis Papantonis, Johannes Kirchmair

**Affiliations:** † Department of Pharmaceutical Sciences, Faculty of Life Sciences, University of Vienna, Josef-Holaubek-Platz 2, 1090 Vienna, Austria; ‡ Christian Doppler Laboratory for Molecular Informatics in the Biosciences, Department for Pharmaceutical Sciences, 27258 University of Vienna, 1090 Vienna, Austria; § Vienna Doctoral School of Pharmaceutical, Nutritional and Sport Sciences (PhaNuSpo), University of Vienna, 1090 Vienna, Austria

## Abstract

Choosing effective molecular representations remains a central challenge in molecular machine learning and is often addressed through costly trial-and-error. While model selection is typically guided solely by predictive performance, analyzing relationships between trained models can reveal additional structure missed by performance metrics. In terms of model similarity metrics, representational alignment techniques, such as centered kernel alignment (CKA), provide a principled framework for comparing models beyond their predictive performance. In this work, we show that representational alignment is fundamentally linked to performance differences between models. For linear regression, we theoretically show that alignment upper-bounds the achievable performance gaps. This result predicts an exclusion zone in which highly aligned models do not exhibit large performance differences, a phenomenon we empirically validate across 661 classification data sets. To make these insights actionable, we introduce the mean minimum class distance (MMCD), a straightforward data set-level statistic that predicts a data set’s position in the alignment–performance difference space. Across 23 molecular representations and ten representative data sets, we find that data sets that produce highly aligned models tend to exhibit low MMCD, suggesting that alignment is strongly shaped by data set-specific structure. Overall, our results indicate that when alignment is low, exploring alternative representations is more likely to improve performance. In contrast, when alignment is high, gains are more effectively achieved by increasing the size of the training data.

## Graphical abstract

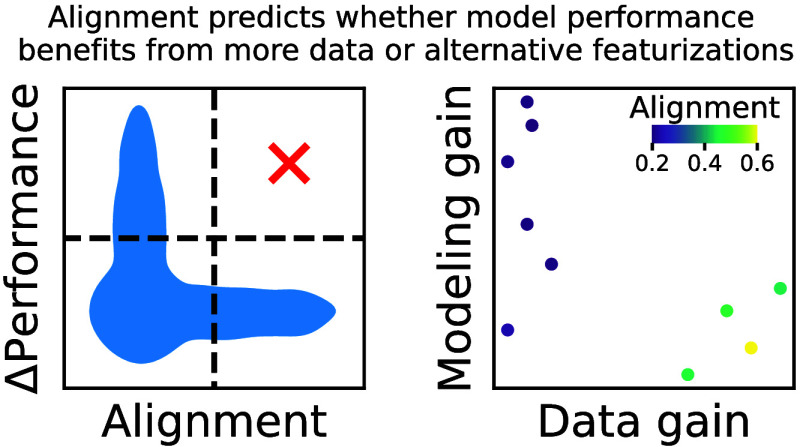

## 1 Introduction

In drug discovery, machine learning (ML) models are increasingly employed to predict molecular properties,^1,2^ prioritize compounds,^3^ and guide decision-making.^4–7^ To this end, models rely on molecular representations to convert chemical structures into machine-readable formats.

One well-established and effective method for encoding molecules for machine learning is to use physicochemical properties (PCs) derived from 2D molecular structures.^8,9^ These molecular descriptors offer a high degree of interpretability, enabling medicinal chemists to directly connect molecular features to hypotheses on pharmacokinetics or efficacy.^10^ The effectiveness of PCs as molecular representations is reflected, for example, by their widespread use in visualizing chemical data sets through dimensionality reduction techniques and toxicity prediction.^11^

Another widely employed method for encoding molecular structures is the use of binary fingerprints (FPs).^12^ These descriptors represent molecules as fixed-length bit vectors by encoding the presence and absence of molecular substructures. Initially developed for rapid similarity searching in chemical databases,^13^ FPs have become a mainstay in machine learning due to their straightforwardness and strong performance in tasks such as bioactivity and ADMET prediction.^9^ On the practical side, while there are numerous ways to design FPs, they tend to perform similarly in downstream applications, as pairwise distances between compounds computed with different strategies are highly correlated^14^ and virtual screening performance is comparable.^15^

Another class of descriptors operates by incorporating 3D structural information, derived from molecular conformers.^8^ Despite their appeal, 3D descriptors rarely outperform 2D descriptors in tasks such as early enrichment in virtual screening, while being more computationally demanding.^16^ However, there is evidence that combining 2D and 3D descriptors can improve scaffold recovery rates.^17^

A common point among the aforementioned molecular representations is that they are all derived using predefined rules and calculations. In contrast, recent ML advances enable learning molecular representations directly from data.^18–23^ Models can derive embeddings from inputs such as molecular graphs or SMILES strings, either via pretraining on large unlabeled data sets^18,20^ or supervised training for specific property predictions.^24^ For example, graph neural networks (GNNs) process molecular graphs using node features (e.g., atom type, neighbor count) and adjacency matrices. The continuous nature of this type of neural network allows for smooth interpolation between molecular structures.^25^ In terms of performance, GNNs often match traditional models, such as random forests using FPs.^24^

Benchmarking studies have shown that performance differences between molecular representations are often within statistical margins, making it challenging to identify a universally superior representation.^9^ However, mounting evidence suggests that comparing models solely by performance can be misleading, as models with similar performance can behave very differently in deployment.^26^ This necessitates the use of alternative metrics to determine model similarity.

An alternative approach to evaluate molecular representations is to directly analyze them at the data set level.^27^ However, this approach does not assume a model and, therefore, is agnostic to the training process, limiting our capacity to improve model design. In contrast, analyzing the internal model representation directly provides fine-grained details about the interplay between the data set, the model, and the optimization process.^28,29^

Representational alignment is a widespread framework for comparing different model representations.^28,30,31^ Theoretical results show that representational alignment provides an upper bound on the additional prediction error incurring when neural networks are combined linearly.^32^ This makes alignment relevant for understanding model compatibility and performance. Furthermore, alignment studies have revealed why models trained under different paradigms, such as self-supervised and supervised learning, often converge to similar embeddings despite their distinct objectives.^33^

In molecular ML, centered kernel alignment (CKA) has demonstrated that regularization improves GNN robustness to size shifts.^34^ A random forest-specific CKA variant revealed that changes in FP radius have a minimal effect on decision boundaries.^35^ CKA has also been applied to validate a self-distillation approach for enhancing performance for low-data scenarios.^36^

While alignment has been used to study robustness^36^ and hyperparameter effects,^35^ it has not been systematically applied to guide the selection of molecular representations. Developing such an approach could allow us to move beyond performance metrics and directly relate internal representation differences to model performance and data set properties.

In this work, we develop a theoretical framework linking representational alignment to differences in the performance of deep learning models. Starting with linear regression, we prove an upper bound on the performance divergence between models trained on distinct representations, showing that alignment constrains the size of these gaps. This result predicts the existence of an exclusion zone where large gaps in model performance do not coexist with high alignment. We then empirically test this hypothesis in classification tasks, finding significant evidence that the exclusion zone holds for binary classification. Finally, we demonstrate that a simple data set statistic is predictive of a data set’s location in the alignment performance-difference space, thereby informing researchers whether collecting additional data or modifying representations is the most promising strategy for improving model performance.

## 2 Methods

### 2.1 Data Sets

This work uses all binary classification data sets of Therapeutics Data Commons (TDC), a widely adopted benchmarking platform for therapeutic machine learning tasks.^37^ Specifically, the collection of data sets used includes 13 data sets related to ADME properties, 12 high-throughput screening data sets, 635 toxicity-related data sets (primarily originating from ToxCast^38^ and Tox21 ^39^), and a single data set on hERG inhibition.

To isolate the effect of model alignment on prediction performance, balanced versions of the data sets were retrieved from TDC via the API. Data sets were capped at a size of 5000 molecules by random sampling to limit the effect of each individual data set on the alignment calculation.

The data sets were standardized using the ChEMBL structure pipeline^40^ with default parameters. Only molecules with a molecular weight below 600 Da were retained to ensure numerical stability during descriptor calculation and model training.

### 2.2 Molecular Representations and Models

All classical descriptors utilized in this study were computed using RDKit.^41^ FP representations were based on Morgan fingerprints^12^ with a radius of 2 and a bit vector length of 2048. PC descriptors comprised all PC descriptors available in RDKit, excluding “Ipc” and “BertzCT” (for which double overflow errors were encountered with large molecules). This resulted in PC feature vectors of length 215. The feature vectors were standard-scaled before their application in machine learning.

In addition to classical descriptors, 12 pretrained graph neural networks and 9 pretrained transformer-based models were employed to generate molecular representations. Graph isomorphism networks were pretrained on tasks including context prediction, edge prediction, atom masking, and mutual information maximization. Transformer-based models were pretrained using autoregressive language modeling. From each pretrained model, molecular representations were extracted from the last three layers as separate feature sets using the molfeat library.^42^

From these 21 pretrained representations, shorthand names were assigned to two specific models from which the final layer was extracted. We refer to the graph neural network pretrained on supervised context prediction using layer 5 (the last layer) as GINC, and to the ChemGPT transformer model with 19 million parameters using layer 24 (the last layer) as ChemGPT.

For each molecular representation, a three-layer multilayer perceptron (MLP) with a hidden dimension of 128 was employed as the predictive model. The MLPs were trained for 30 epochs using the Adam optimizer^43^ with a learning rate of 10^–4^, a weight decay of 5 ·10^–4^, the ReLU activation function, and a batch size of 1000.

### 2.3 Representational Alignment

The representational alignments between models were calculated using the embeddings extracted from the final hidden layer of each MLP. Furthermore, the alignment between each model pair was evaluated using a fixed, shared data set containing all molecules appearing in the different 661 training data sets. The shared data set contains 45,134 unique molecules and 23,781 unique Bemis–Murcko scaffolds (a more detailed description is provided in SI Section A).

The representational alignment was assessed using CKA,^28^ which is arguably the most widely used measure of model similarity, partly because it shares a series of properties with neural networks (invariance to orthogonal transformations and isotropic scaling).^28,30,31^ In Section 3.1, a theoretical argument that further supports the use of CKA as an alignment function (see Proposition 1) is provided. CKA is defined as

1 $$a\mathrm{lign}\left(X,Z\right)≔{\mathrm{CKA}}_{\mathrm{linear}}\left(X,Z\right)=\frac{∥X^{T}H_{n}Z{∥}_{F}^{2}}{∥X^{T}H_{n}X{∥}_{F}∥Z^{T}H_{n}Z{∥}_{F}}$$

where *X* and *Z* are the feature matrices coming from the different models and $H_{n}=I_{n}-\frac{1}{n}11^{T}$ is the centering matrix. CKA is a normalized version of a measure of dependence between two random variables, aimed at estimating the sum of the squared singular values of the cross-covariance operator.^28,30,44^

## 3 Results

We investigate performance differences between models using representational alignment as a guiding framework. We begin with a theoretical analysis that shows alignment between two representations (eq 1) constrains the maximum difference in model predictions under linear regression. This constraint creates an exclusion zone where high alignment prevents substantial differences in predictive performance. We then empirically examine whether this relationship holds for binary classification tasks, which are particularly relevant to the field of drug discovery. Next, we demonstrate that a simple data set characteristic enables low-cost prediction of a data set’s approximate position in the alignment-performance difference (APD) space. To assess robustness, we analyze a smaller set of data sets with a broader range of representations and confirm that the APD pattern remains consistent. Finally, we illustrate the practical utility of these findings: alignment can indicate whether performance gains are more likely to come from collecting additional data or from exploring alternative molecular representations, providing a principled guide for modeling decisions.

### 3.1 Performance Difference is Bound by Model Alignment

In this section, we formalize the intuition that more aligned representations tend to produce more similar outputs. To this end, we state the following proposition (proof in SI Section B, Proposition 1), which shows that as the alignment between the feature matrices X and Z increases, the performance difference between linear regression models (i.e., multivariate linear regression using ordinary least-squares) trained on each of them decreases. Interestingly, a similar result was reported in ref 32., where the excess risk of linearly merging two neural networks (i.e., the mean-square-error discrepancy between the best merged model and its nonmerged counterpart) is bounded in terms of kernel alignment. Our results differ in framing but support the same insight: alignment constrains the extent to which models can disagree.

**Proposition 1.** *Let X and Z be centered feature matrices, and y be a normalized response vector. Moreover, let W and V be the coefficients obtained by linearly regressing X and Z to y respectively, then*

$$∥\mathrm{XW}-y{∥}_{2}-∥\mathrm{ZV}-y{∥}_{2}\leq \sqrt{\mathrm{rank}X+\mathrm{rank}Z-2\cdot \mathrm{align}\left(X,Z\right)}$$

Although Proposition 1 is stated for linear regression models, it has direct implications for neural networks used in regression tasks. This is because, in essence, neural networks can be viewed as a composition of an encoder and a linear readout layer,^29^ which is equivalent to performing linear regression over the representations extracted from the final hidden layer. Hence, alignment in internal representations influences downstream performance by constraining the performance gap between models.

Importantly, Proposition 1 implies that greater alignment between models yields tighter bounds on their performance differences, assuming all other factors remain constant. A direct consequence of this observation is that large performance gaps are only possible when the alignment between representations is low, giving rise to what we call an exclusion zone, i.e., the high alignment-high performance gap region, which is inaccessible due to alignment constraints. In practical terms, this means that even if models are trained on vastly different input features, as long as their internal representations are aligned, they should yield similar outcomes.

Previous work argues that a good measure of alignment should exhibit a correlation between representational alignment and predicted outcomes.^45^ However, it has been found that the strength of this pattern is data set-dependent.^46^

The proposed exclusion zone provides a complementary view on the relationship between alignment and performance differences that might persist across data sets. It is worth noting that this phenomenon has been implicitly already observed in prior work: for example, in ref 46, Figure 8, the strength of correlation between performance differences and alignment varies across data sets; however, there are no model pairs with high performance differences and high alignment displayed in that figure.

Returning to our Proposition 1, we observe that the bound involves the ranks of the feature matrices, which may potentially dominate the right-hand side of the inequality. Although this is an indication that the bound may be loose, it motivates a deeper investigation of the alignment–performance difference relationship. This inquiry is especially relevant because representations in later layers of neural networks often lie on low-dimensional manifolds,^47^ effectively reducing the rank of the feature matrix and, in turn, potentially increasing the predictive value of alignment for performance differences.

### 3.2 Exclusion Zone Extends Beyond Theory

While Proposition 1 addresses performance differences in regression problems, cheminformatics primarily involves classification tasks (e.g., predicting ADMET properties) for which no comparable theoretical results exist. To bridge this gap, we present an empirical investigation to examine whether similar principles govern classification performance. Specifically, we analyze a series of binary classification tasks from the TDC initiative,^37^ aiming to connect the theoretical regression setting of Proposition 1 with real-world molecular classification problems.

Figure 1a shows the relationship between representational alignment and differences in model performance (APD analysis), measured using the difference in the Matthew correlation coefficient (MCC).^48^ Each point represents the average alignment and average performance difference across all model combinations based on the PC, FP, ChemGPT, and GINC featurizations (the nonaggregated APD analysis is provided in SI Section C). The figure shows a clear trend in representational alignment, with considerable variability in model performance at low alignment. However, as alignment increased, model performance became more consistent. This trend indicates that even in a task-specific setting, alignment in internal representations constrains differences in predictive performance.

**Figure 1:**
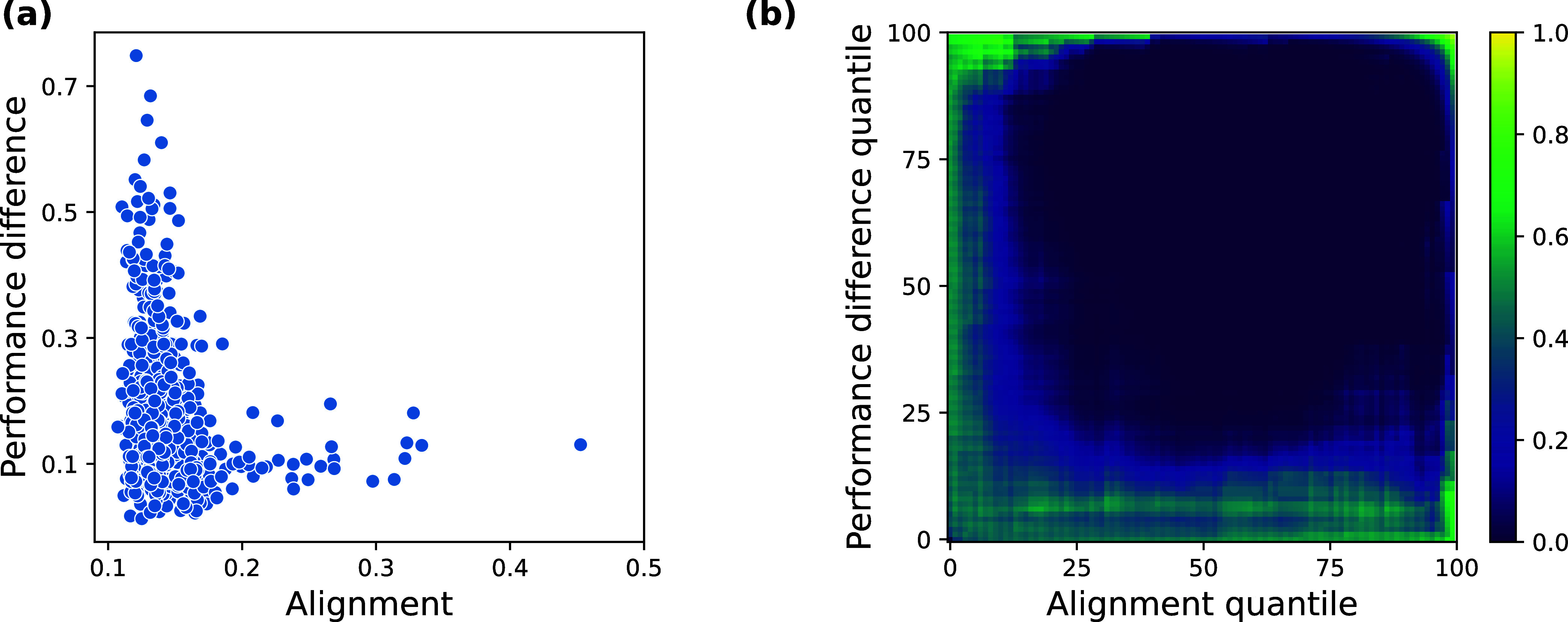
Comparison of performance difference and alignment for 661 data sets. (a) Average alignment of models based on PC, FP, ChemGPT, and GINC plotted against corresponding average performance differences (measured by MCC). (b) Binomial test results for varying quantile thresholds, with color intensity indicating p-value magnitude (darker regions correspond to lower p-values).

Binomial testing revealed a coherent cluster of neighboring cells (the dark blue region in Figure 1b) with significantly low p-values concentrated in the high-alignment region (above the 20th percentile) across a broad range of performance differences (above the 20th percentile). This indicates that each of these cells is significantly underpopulated with data sets. Moreover, the spatial coherence of this cluster, rather than scattered isolated cells, suggests a systematic exclusion pattern rather than random fluctuations in data density. Note that the small cluster of high p-values in the upper-right corner of Figure 1b is an artifact of finite support. In our experiments, there are no model combinations in the high-alignment, high-performance-difference regime. However, if there were no relationship between alignment and performance differences, this region would also be sparsely populated. Accordingly, the p-values are elevated but should not be interpreted as evidence against the exclusion pattern.

To assess whether the observed clustering pattern could arise by chance and to account for multiple comparisons, we employed a widely used cluster-based permutation test.^49^ This test assesses the likelihood of such a cluster arising, assuming there is no association between performance differences and alignment. The p-value of 0.0001 that resulted from this test confirms that the observed pattern is unlikely the result of random variation. The details of this approach are provided in SI Section D.

The significant clustering pattern provides empirical evidence for an exclusion zone that closely matches the theoretical predictions derived from our linear regression analysis, demonstrating that alignment between models also constrains the performance difference in classification problems.

### 3.3 Data Set Features Predict Alignment–Performance Difference

In the previous section, we showed that alignment naturally constrains performance differences between models. To translate this insight into actionable guidance for modeling decisions, the ability to predict a data set’s position in the APD space without running all experiments is essential. Two concepts required for the following discussion are separability and margin. Separability describes whether two subsets can be separated from each other, while margin is the minimum distance between a decision boundary and any training data point. These two concepts are related with alignment as shifting the margin between two separable sets can lead to lower alignment while maintaining performance.^50^ At the same time, margin between positive and negative classes influence machine learning models’ generalization capabilities, as shown in a recent antibody–antigen binding study.^51^

Motivated by the finding that data set geometry can influence predictive performance and alignment, we hypothesize that the margin between the classes in a data set can be used to predict its position in the APD space. Specifically, when instances from different classes are close (i.e., the margins are small), models trained on this data set may learn similar decision boundaries, resulting in high alignment between them. This rationale is illustrated in Figure 2. Conversely, when the classes are well-separated, the task becomes less well-defined (i.e., many decision boundaries fit the training data but not necessarily the test data), potentially resulting in lower alignment across models.

**Figure 2:**
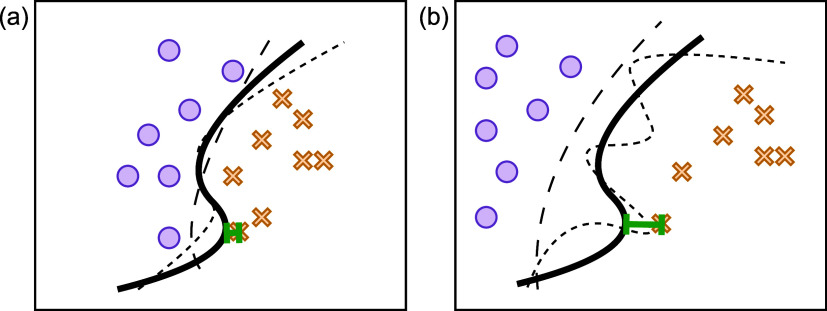
Illustration of the hypothesized effect that margin has on alignment in binary classification (samples from the two classes are represented as blue circles and orange crosses; the bold black line represents the true decision boundary; the dashed lines represent possible learned decision boundaries): (a) a small margin (illustrated by the green indicator) leads to more similar decision boundaries and, hence, high alignment; (b) a large margin allows varying decision boundaries, leading to low alignment.

To operationalize the distance between classes as a proxy for predicting the underlying data set’s position in the APD space, the first step was to quantify the proximity between the classes concretely. To this end, we introduced a simple data set-level descriptor, the mean minimum class distance (MMCD), defined as the minimum average distance between a compound of one class and all compounds of the other class. Ideally, the distance used to calculate the MMCD should reflect what is important in a given task. For example, if molecular weight is known to be important (e.g., solubility), one could consider it to calculate the distance between two compounds. Molecular structure plays a decisive role in many cheminformatics tasks, including virtual screening and property prediction.^9,16^ Hence, we calculate the MMCD using the Tanimoto distance between molecules represented as FPs in our experiments due to its widespread use, its intuitiveness, and speed of calculation, yielding a single value that characterizes how close two compound classes are.

The second step was to assess the effectiveness of MMCD in predicting a data set’s position in the APD space. For our purposes, it was sufficient to predict which of the following three regions a data set falls into: (1) low alignment with low performance difference, (2) high alignment with low performance difference, or (3) low alignment with high performance difference. Note that we did not consider the region of high alignment with a high performance difference, as this corresponds to the exclusion zone discussed in the previous sections.

To associate individual data sets with one of the three regions, we modeled region assignment as a clustering problem, utilizing 495 of 661 data sets to define the clusters and holding out the remaining 166 data sets for the validation of our approach. Specifically, we used k-means to cluster the training data, deciding the optimal number of clusters by maximizing the silhouette score (a measure of cluster quality^52^). Eventually, k = 3 clusters achieved the best score (peak value 0.51 ± 0.00, averaged across 100 initializations; details in SI Section E). Figure 3a,b visualize the obtained cluster labels and MMCDs for all training data sets.

**Figure 3:**
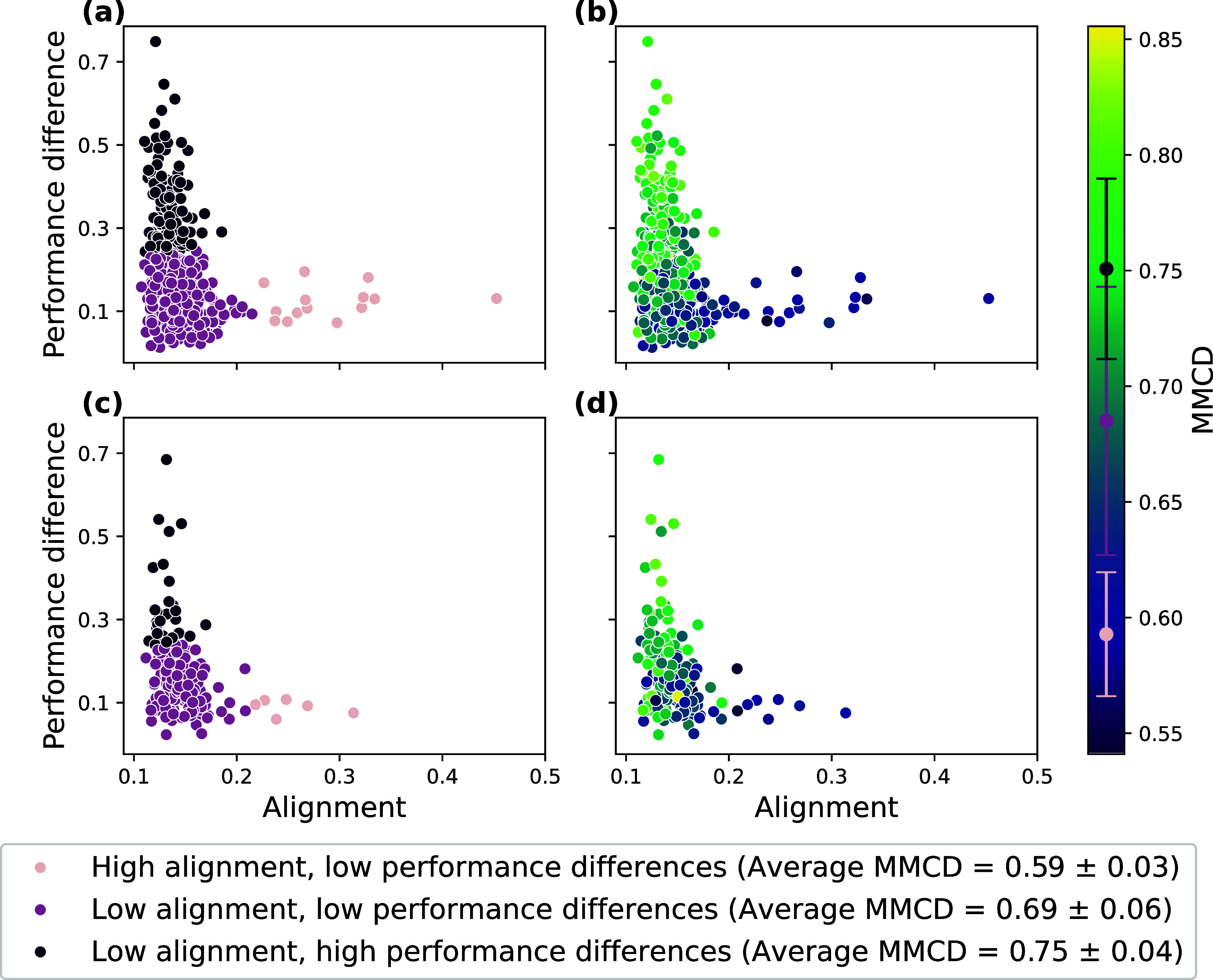
APD spaces of the data sets used to define clusters (panels (a) and (b)) and the data sets used to assess MMCDs predictive performance (panels (c) and (d)). Individual data sets are colored according to the label obtained from k-means clustering (panels (a) and (c)) or according to the MMCD (panels (b) and (d)). Average MMCD values for the three relevant regions of the APD space are listed with their standard deviation in the legend and depicted in the colorbar.

Having partitioned the 495 data sets into separate clusters, we needed a single value to indicate each cluster’s overall MMCD. We obtained this indicator by averaging the MMCD values across all data sets in a given cluster. This process yielded 3 values, which we used to assign the remaining 166 data sets to a cluster by computing the MMCD for each test data set and assigning it to the cluster with the closest average MMCD. By comparing the labels derived from the MMCD (Figure 3d) with those assigned using distances in the original APD space (Figure 3c), we achieved a balanced accuracy of 0.73, demonstrating the predictive power of this approach.

### 3.4 Exclusion Zone Persists Across Representations

In the previous sections, we reported the observation of an exclusion zone in APD space using four featurization strategies (PC, FP, ChemGPT, and GINC). We also found that data sets with low MMCD (around 0.6) tended to have high alignment, whereas those with higher MMCD generally showed low alignment. However, it remains to be tested whether these findings hold across different featurization strategies or are an artifact of the four strategies explored so far.

In addition to the RDKit implementations of Morgan FPs and PCs already considered, we included a set of diverse learned representations. In particular, we focused on models with varying pretraining objectives, as differences in training objectives have been identified as a key factor driving alignment.^46^ More specifically, we selected 12 GNNs with 4 pretraining objectives (context prediction, edge prediction, atom masking, and mutual information maximization) and 9 chemical language models (a detailed description is provided in the Methods section).

In total, our selection of featurization strategies resulted in 23 distinct representations, for which we then conducted an analysis similar to that presented in Section 3.2. However, since it was computationally infeasible to consider all 661 data sets, we selected ten biologically diverse data sets that are chemically similar to the full data set collection (a detailed description is provided in SI Section F).

Figure 4 shows the performance differences and alignments observed across the 253 model pairs per data set in blue. In addition, the per-data set aggregates (generated by averaging over all APD pairs that correspond to the same underlying data set) are shown as large points with error bars indicating the variability across model pairs from this aggregation. Consistent with the experiments in Section 3.2, alignment again shaped performance differences between models. Notably, only 0.7% of model pairs differed in performance by more than 1 minus their alignment score, highlighting how alignment constrains performance variability. In addition, as alignment increased, the variance of performance differences steadily declined, from 0.038 in the lowest-alignment regime to 0.007 in the highest-alignment regime (a visualization is provided in SI Section G). This result suggests that the exclusion zone persists across additional representations.

**Figure 4:**
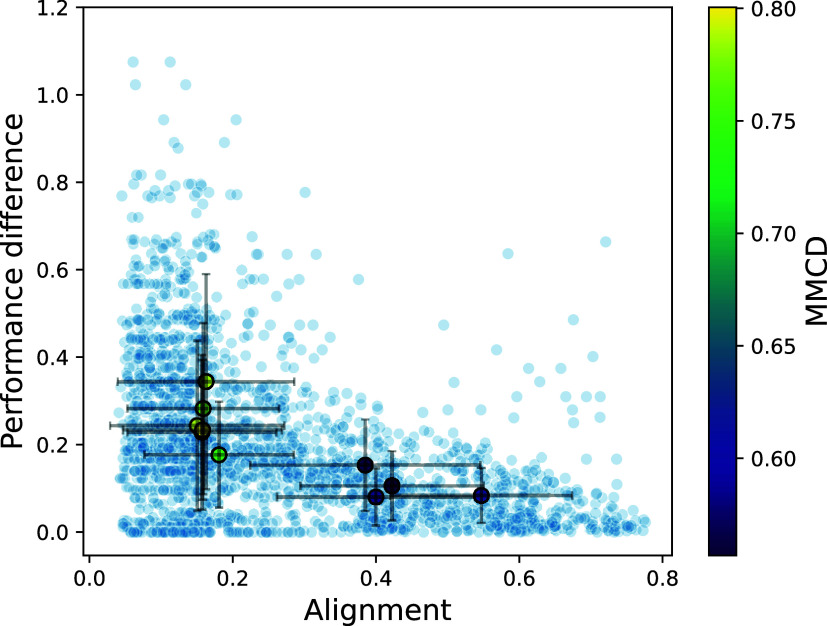
Each small blue dot represents one model pair among 23 featurization strategies, plotting model alignment versus the corresponding performance difference. The data set-level aggregates are shown as larger dots (mean) with error bars based on the standard deviation and are colored according to the MMCD.

In Figure 4, we colored the data set-level aggregates by MMCD (a measure of distance between the two classes, defined in Section 3.3 that seems predictive of a data set’s location in APD space). Examining the data set-level aggregates revealed four data sets with high average alignment between models (exceeding 0.3): the cytochrome P450 data sets CYP1A2, CYP2D6, CYP3A4, and the hERG channel inhibition data set.

Despite the high variation in alignment (with an overall average standard deviation of 0.14), high-alignment data sets showed MMCDs below 0.61. In contrast, low-alignment data sets showed notably larger MMCDs, exceeding 0.75. This pattern reinforces that the MMCD can be used to predict a data set’s position in the APD space. The results raise the question of whether alignment is primarily determined by data set-specific factors.

### 3.5 Alignment Reveals the Drivers of Model Performance

In this section, we provide practical guidelines for modeling decisions. Specifically, we investigate whether model alignment can serve as an indicator of whether performance gains are more likely to come from expanding training data or from exploring alternative representations.

#### 3.5.1 Model Performance Is Driven by Featurization for Low Alignment Data Sets

We begin by considering a common scenario in cheminformatics, where several molecular representations have been used to train and evaluate models, and we ask whether exploring additional representations is likely to improve performance. In our setting, we define meaningful improvement as an increase of at least C% over the best-performing existing model. Formally, given n trained models with performances *m*_1_, *m*_2_, ···, *m*_*n*_, we are interested in *p*_*better*_ = *p*(*m*_*n*+1_ > *C* · max­(*m*_1_,···, *m*_*n*_) |*m*_1_,···, *m*_*n*_).

We estimate the probability of meaningful performance improvement *p*_better_ using a simulation-based approach. For each data set in Section 3.4, we randomly select *n* of the 23 available models, record the maximum performance among them and average pairwise alignment, then draw an additional model and assess whether its performance exceeds the current maximum by at least C%. We repeat this procedure 10,000 times, varying the number of reference models from 3 to 7 and the relative improvement threshold C from 10 to 50%.

Figure 5 shows the probability that the next model improved upon the best of the previous n models by at least C%, grouped by alignment. As expected, larger improvements were in general less likely, with almost all curves following a nonincreasing trend. Similarly, the more models that had already been evaluated, the less likely it was that an additional one would outperform them. Across panels, as *n* increased, the curves consistently attained lower values.

**Figure 5:**
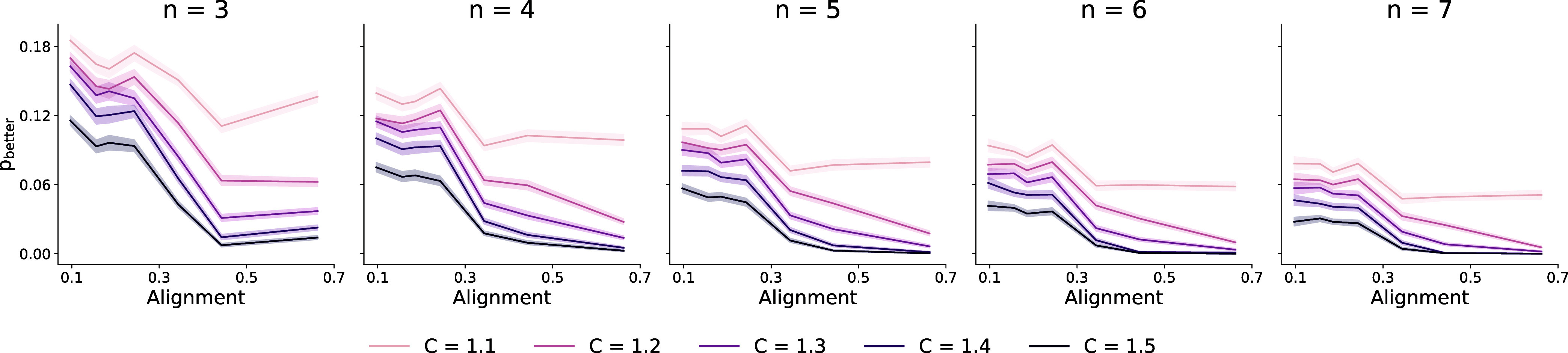
Probability *p*_better_ for an additional model to outperform the current best model by at least C% after having evaluated *n* models. *p*_better_ dropped with increasing number *n* of previously evaluated models, larger minimum improvement requirements *C*, and alignment.

Shifting our focus to alignment, we observed that the probability of improvement decreased with greater alignment. Gains that were moderately likely in low-alignment settings became rare in high-alignment settings. Notably, averaging across all *n* and *C* combinations, the probability of improvement dropped from 11% in low-alignment regimes to below 3% in high-alignment ones. In practice, this suggests that when existing models show high alignment, further exploration of alternative representations is unlikely to yield meaningful gains.

#### 3.5.2 High Alignment Data Sets Improve with More Data

In the previous section, we showed that performance improvements from alternative featurization strategies are more likely in low-alignment settings. To complement this analysis, we now ask the opposite question: is model performance likely to improve by collecting more data points, in particular when the alignment between models is high?

To address this question, we use the same data sets as in Section 3.4 and simulate incremental data collection by training models on subsets of the available training data, going from 75 to 95% in 5% increments. To isolate the effect of data set size from representation choice, for each data set, we use a randomly selected representation out of the 23 representations (data set name to representation mapping is reported in SI Section H), based on Section 3.4, repeating each training run 30 times to mitigate sampling randomness. In addition, to quantify performance scaling with respect to data set size, we compute the Spearman rank correlation between the amount of training data and model performance. Finally, we are interested in testing whether alignment predicts scaling behavior, which, however, presents a great challenge with our current setup. This is because running the 6496 alignment calculations from the previous sections required us to repeatedly load embeddings due to RAM limitations, with the resulting I/O dominating the computational costs. This means that although alignment calculations are computationally inexpensive, the entire pipeline is not. For the incremental data collection simulation, we would need 111750 alignment calculations, which was infeasible. Hence, we opted for the already computed alignment values from the 23 representations in Section 3.4 as a substitute to assess whether alignment predicts scaling behavior.

Table 1 shows the Spearman rank correlation between the amount of training data used for model training and performance for ten data sets from Section 3.4. The cytochrome P450 inhibition data sets and the hERG data set exhibited distinctly higher ranking correlations (*r*_*s*_ > 0.38), indicating a strong positive relationship between the amount of training data used for model training and model performance. In contrast, for all other data sets, correlations remained weak (*r*_*s*_ < 0.10) with confidence intervals that include 0.

**Table 1:** Spearman Rank Correlation between the Amount of Training Data Used for Model Training and Performance with 95% Confidence Intervals from Bootstrapping

**data set name**	***r***_***s***_	**95% CI**	**data set name**	***r***_**s**_	**95% CI**
CYP2D6	0.57	[0.46, 0.68]	TF	0.05	[−0.10, 0.22]
CYP3A4	0.46	[0.33, 0.57]	SULT2A	0.06	[−0.11, 0.21]
CYP1A2	0.51	[0.39, 0.62]	PTPN11	0.05	[−0.10, 0.21]
STK	0.01	[−0.15, 0.19]	5-HT1	0.10	[−0.04, 0.26]
HepG2	0.01	[−0.14, 0.19]	hERG	0.38	[0.25, 0.50]

To assess the role of alignment, we compared the average alignment between the low-performance increase and high-performance increase groups. Data sets showing an increase in model performance with more training data exhibited a mean alignment of 0.46 ± 0.07, whereas those with inconsistent gains had a mean alignment of 0.18 ± 0.01. In practice, this suggests that when existing models show high alignment, collecting additional training data is likely to improve performance. On the other hand, when alignment is low, additional training data may not lead to better models. Exploring alternative representations may be a more fruitful strategy in this scenario.

## 4 Conclusions

In this work, we investigated the relationship between representational alignment and performance differences in molecular machine learning applications. We began by considering regression models, establishing a theoretical link between alignment and performance differences. This led to the identification of an exclusion zone in the high-difference, high-alignment regime, which we empirically validated across more than 600 classification data sets. We further demonstrated the robustness of this phenomenon by evaluating 23 diverse featurization strategies, showing that the exclusion zone persists across a broad range of molecular representations and learning settings.

Following up on this result, we defined MMCD, a straightforward data set-level aggregate statistic that reliably predicts a data set’s location in the alignment–performance difference space. This result highlights the potential importance of data set-specific properties in shaping representational alignment between models, an aspect that has received little attention in prior work and warrants further investigation.

Finally, we translated these findings into actionable guidance for model development. Our results indicated that in low-alignment regimes, exploring alternative featurizations is more likely to improve performance, while in high-alignment regimes, gains are more effectively achieved by increasing the size of the training set.

In cheminformatics, labeled data is often costly to obtain while the performance differences between models may be modest. Under these conditions, deciding whether to explore additional models or acquire more training data is critical. Our results indicate that representational alignment can serve as a practical guide in making this decision.

Here, we focus on neural networks but related frameworks may, in principle, apply to other model classes. However, going beyond neural networks can introduce several challenges. First, it is often unclear how to define the relevant embedding or the associated kernel for comparisons within one model class.^35^ Cross-class comparisons might pose another challenge, as different kernels can have different eigenspectrum characteristics, which might distort measures of representational alignment.

Taken together, our findings suggest that representational alignment provides a unifying lens for understanding both empirical performance differences and their inherent limitations in molecular machine learning. Looking forward, this perspective opens avenues for studying how data set characteristics, inductive biases, and training dynamics jointly shape the performance improvements as models grow increasingly complex. More broadly, this work highlights that alignment-based analyses provide a principled framework for comparing models beyond benchmark scores alone, establishing a complementary axis to assess progress, diversity, and redundancy in molecular representations.

## Supplementary Material



## Data Availability

This study used publicly available data from the Therapeutics Data Commons (TDC).^37^ All data can be accessed programmatically through the TDC Python package (version 0.4.1). The source code of the approach presented in this work is available from https://github.com/molinfo-vienna/pc_fp_align_paper/.

## Notes

Further details on the analysis of shared evaluation data set, proof of Proposition 1, nonaggregated APD analysis for 661 data sets with four representations, detailed description of cluster-based permutation tests, discussion on the selection of number of centroids for analyzing the relationship between MMCD and alignment, discussion on data set selection for more in depth experiments with respect to chemical similarity of the entire data set collection and biological diversity, and discussion on how variance of performance differences decreases with increasing alignment, and description of the selected featurization strategies for Section 3.5.2 (PDF)

## References

[1] Li Z., Jiang M., Wang S., Zhang S. (2022). Deep learning methods
for molecular representation and property prediction. Drug Discovery Today.

[2] Mayr A., Klambauer G., Unterthiner T., Hochreiter S. (2016). DeepTox: Toxicity
prediction using deep learning. Environ. Sci..

[3] Schneider G. (2018). Automating
drug discovery. Nat. Rev. Drug Discovery.

[4] Vamathevan J., Clark D., Czodrowski P., Dunham I., Ferran E., Lee G., Li B., Madabhushi A., Shah P., Spitzer M., Zhao S. (2019). Applications
of machine learning in drug discovery and development. Nat. Rev. Drug Discovery.

[5] Dara S., Dhamercherla S., Jadav S. S., Babu C. M., Ahsan M. J. (2022). Machine
learning in drug discovery: a review. Artif.
Intell. Rev..

[6] Boldini D., Friedrich L., Kuhn D., Sieber S. A. (2024). Machine learning
assisted hit prioritization for high throughput screening in drug
discovery. ACS Cent. Sci..

[7] Pillai N., Dasgupta A., Sudsakorn S., Fretland J., Mavroudis P. D. (2022). Machine
learning guided early drug discovery of small molecules. Drug Discovery Today.

[8] Consonni, V. ; Todeschini, R. Molecular Descriptors for Chemoinformatics; Wiley-VCH: Weinheim, Germany, 2009.

[9] Deng J., Yang Z., Wang H., Ojima I., Samaras D., Wang F. (2023). A systematic study of key elements
underlying molecular property
prediction. Nat. Commun..

[10] Daina A., Zoete V. (2016). A BOILED-Egg to predict
gastrointestinal absorption and brain penetration
of small molecules. ChemMedChem.

[11] Clemons P. A., Wilson J. A., Dančík V., Muller S., Carrinski H. A., Wagner B. K., Koehler A. N., Schreiber S. L. (2011). Quantifying
structure and performance diversity for sets of small molecules comprising
small-molecule screening collections. Proc.
Natl. Acad. Sci. U.S.A..

[12] Rogers D., Hahn M. (2010). Extended-connectivity fingerprints. J. Chem.
Inf. Model..

[13] Christie B.
D., Leland B. A., Nourse J. G. (1993). Structure searching in chemical databases
by direct lookup methods. J. Chem. Inf. Comput.
Sci..

[14] Boldini D., Ballabio D., Consonni V., Todeschini R., Grisoni F., Sieber S. A. (2024). Effectiveness of molecular fingerprints
for exploring the chemical space of natural products. J. Cheminf.

[15] Riniker S., Landrum G. A. (2013). Open-source platform
to benchmark fingerprints for
ligand-based virtual screening. J. Cheminf.

[16] Hu G., Kuang G., Xiao W., Li W., Liu G., Tang Y. (2012). Performance
evaluation of 2D fingerprint and 3D shape similarity
methods in virtual screening. J. Chem. Inf.
Model..

[17] Fan N., Hirte S., Kirchmair J. (2022). Maximizing
the performance of similarity-based
virtual screening methods by generating synergy from the integration
of 2D and 3D approaches. Int. J. Mol. Sci..

[18] Hu, W. ; Liu, B. ; Gomes, J. ; Zitnik, M. ; Liang, P. ; Pande, V. ; Leskovec, J. Proceedings of the 8th International Conference on Learning Representations; Addis Ababa: Ethiopia, 2020; Strategies for pre-training graph neural networks, https://openreview.net/.

[19] Wang, S. ; Guo, Y. ; Wang, Y. ; Sun, H. ; Huang, J. Computational biology and health informatics; Association for Computing Machinery: New York, NY, USA, 2019; SMILES-BERT: Large scale unsupervised pre-training for molecular property prediction.

[20] Chithrananda, S. ; Grand, G. ; Ramsundar, B. ChemBERTa: large-scale self-supervised pretraining for molecular property prediction. 2000; https://arxiv.org/abs/2010.09885.

[21] Frey N. C., Soklaski R., Axelrod S., Samsi S., Gomez-Bombarelli R., Coley C. W., Gadepally V. (2023). Neural scaling of deep chemical models. Nat. Mach. Intell..

[22] Rong, Y. ; Bian, Y. ; Xu, T. ; Xie, W. ; Wei, Y. ; Huang, W. ; Huang, J. Proceedings of the 34th International Conference on Neural Information Processing Systems; Curran Associates Inc.: Virtual, 2020; Self-supervised graph transformer on large-scale molecular data.

[23] Bagal V., Aggarwal R., Vinod P., Priyakumar U. D. (2022). MolGPT:
molecular generation using a transformer-decoder model. J. Chem. Inf. Model..

[24] Yang K., Swanson K., Jin W., Coley C., Eiden P., Gao H., Guzman-Perez A., Hopper T., Kelley B., Mathea M., Palmer A., Settels V., Jaakkola T., Jensen K., Barzilay R. (2019). Analyzing
learned molecular representations for property
prediction. J. Chem. Inf. Model..

[25] Boyar, O. ; Hanada, H. ; Takeuchi, I. Conditional latent space molecular scaffold optimization for accelerated molecular design. TMLR 2025, https://openreview.net/forum?id/KhxVc9RBJv.

[26] D’Amour A. (2022). Underspecification presents
challenges for credibility in modern
machine learning. J. Mach. Learn. Res..

[27] Rottach F., Schieferdecker S., Eickhoff C. (2025). The topology of molecular representations
and its influence on machine learning performance. J. Cheminf..

[28] Kornblith, S. ; Norouzi, M. ; Lee, H. ; Hinton, G. Proceedings of the 36th International Conference on Machine Learning; PMLR: Long Beach, USA, 2019; Similarity of neural network representations revisited.

[29] Alain, G. ; Bengio, Y. Proceedings of 5th International Conference on Learning Representations; https://openreview.net/: Toulon, France, 2017; Understanding intermediate layers using linear classifier probes.

[30] Klabunde M., Schumacher T., Strohmaier M., Lemmerich F. (2025). Similarity
of neural network models: A survey of functional and representational
measures. ACM Comput. Surv..

[31] Sucholutsky, I. Getting aligned on representational alignment. 2024; https://arxiv.org/abs/2310.13018.

[32] Insulla, F. ; Huang, S. ; Rosasco, L. Proceedings of the 13th International Conference on Learning Representations; https://openreview.net/: Singapore, 2025; Towards a learning theory of representation alignment.

[33] Luthra, A. ; Mishra, P. ; Galanti, T. On the alignment between supervised and self-supervised contrastive learning. 2025; https://arxiv.org/abs/2510.08852.

[34] Buffelli, D. ; Lio, P. ; Vandin, F. Proceedings of the 35th International Conference on Neural Information Processing Systems; Curran Associates Inc.: Cambridge, USA, 2022; SizeShiftReg: a regularization method for improving size-generalization in graph neural networks.

[35] Welsch M., Hirte S., Kirchmair J. (2024). Deciphering
molecular embeddings
with centered kernel alignment. J. Chem. Inf.
Model..

[36] Palmacci V., Nahal Y., Welsch M., Engkvist O., Kaski S., Kirchmair J. (2025). E-GuARD: expert-guided augmentation for the robust
detection of compounds interfering with biological assays. J. Cheminf.

[37] Huang, K. ; Fu, T. ; Gao, W. ; Zhao, Y. ; Roohani, Y. ; Leskovec, J. ; Coley, C. W. ; Xiao, C. ; Sun, J. ; Zitnik, M. Thirty-fifth Conference on Neural Information Processing Systems Datasets and Benchmarks Track; https://openreview.net/: Virtual, 2021; Therapeutics Data Commons: Machine learning datasets and tasks for drug discovery and development.

[38] Richard A. M., Judson R. S., Houck K. A. (2016). ToxCast chemical landscape:
Paving the road to 21st century toxicology. Chem. Res. Toxicol..

[39] Richard A. M., Huang R., Waidyanatha S. (2021). The Tox21 10K compound
library: Collaborative chemistry advancing toxicology. Chem. Res. Toxicol..

[40] Bento A. P., Hersey A., Félix E., Landrum G., Gaulton A., Atkinson F., Bellis L. J., De Veij M., Leach A. R. (2020). An open
source chemical structure curation pipeline using RDKit. J. Cheminf.

[41] Landrum, G. A. RDKit: Open-source cheminformatics. https://www.rdkit.org/..

[42] Noutahi, E. ; Wognum, C. ; Mary, H. ; Hounwanou, H. ; Kovary, K. M. ; Gilmour, D. ; thibaultvarin r; Burns, J. ; St-Laurent, J. ; t; DomInvivo; Maheshkar, S. rbyrne momatx datamol-io/molfeat. 2023; 10.5281/zenodo.8373019.

[43] Kingma, D. P. ; Ba, J. Adam: A method for stochastic optimization. 2014; https://arxiv.org/abs/1412.6980.

[44] Gretton, A. ; Bousquet, O. ; Smola, A. ; Schölkopf, B. Algorithmic Learning Theory; Springer: Berlin, Heidelberg, 2005; Measuring Statistical Dependence with Hilbert-Schmidt Norms.

[45] Klabunde, M. ; Wald, T. ; Schumacher, T. ; Maier-Hein, K. ; Strohmaier, M. ; Lemmerich, F. Proceedings of the 13th International Conference on Learning Representations; https://openreview.net/: Singapore, 2025; ReSi: A comprehensive benchmark for representational similarity measures.

[46] Ciernik, L. ; Linhardt, L. ; Morik, M. ; Dippel, J. ; Kornblith, S. ; Muttenthaler, L. Proceedings of the 42nd International Conference on Machine Learning; https://openreview.net/: Vancouver, Canada, 2025; Objective drives the consistency of representational similarity across datasets.

[47] Ansuini, A. ; Laio, A. ; Macke, J. H. ; Zoccolan, D. Advances in Neural Information Processing Systems; Curran Associates Inc.: Vancouver, Canada, 2019; Intrinsic dimension of data representations in deep neural networks.

[48] Chicco D., Warrens M. J., Jurman G. (2021). The Matthews Correlation
Coefficient
(MCC) is more informative than Cohen’s Kappa and Brier Score
in binary classification assessment. IEEE Access.

[49] Maris E., Oostenveld R. (2007). Nonparametric
statistical testing of EEG- and MEG-data. Journal
of Neuroscience Methods.

[50] Davari, M. ; Horoi, S. ; Natik, A. ; Lajoie, G. ; Wolf, G. ; Belilovsky, E. ; NeurIPS, M. L. NeurIPS ML Safety Workshop; Curran Associates Inc.: New Orleans, Louisiana, United States, 2022; Deceiving the CKA Similarity Measure in Deep Learning.

[51] Ursu E., Minnegalieva A., Rawat P., Chernigovskaya M., Tacutu R., Sandve G. K., Robert P. A., Greiff V. (2025). Training data
composition determines machine learning generalization and biological
rule discovery. Nat. Mach. Intell..

[52] Rousseeuw P. J. (1987). Silhouettes:
A graphical aid to the interpretation and validation of cluster analysis. J. Comput. Appl. Math..

