# Supplementary material for: Relating Model Performance to Embedding Distributions in Molecular Machine Learning
Source: J Chem Inf Model. 2026 Apr 22;66(9):5125–34. doi: 10.1021/acs.jcim.6c00218 (PMC13169369; doi:10.1021/acs.jcim.6c00218)
Supplement: Supplementary file 1 [file ci6c00218_si_001.pdf]

Supporting information

Relating model performance to embedding distributions in molecular machine learning

Matthias Welsch,[†,‡,¶] Ellena Jiang,[†] Ioannis Papantonis,[†] and Johannes Kirchmair[*,†,‡]

†*Department of Pharmaceutical Sciences, Faculty of Life Sciences, University of Vienna, Josef-Holaubek-Platz 2, 1090 Vienna, Austria*

‡*Christian Doppler Laboratory for Molecular Informatics in the Biosciences, Department for Pharmaceutical Sciences, University of Vienna, 1090 Vienna, Austria*

¶*Vienna Doctoral School of Pharmaceutical, Nutritional and Sport Sciences (PhaNuSpo), University of Vienna, 1090 Vienna, Austria*

E-mail: johannes.kirchmair@univie.ac.at

Contents

A Diversity of the shared evaluation data set **S3**

B Proof of Proposition 1 **S3**

C Non-aggregated APD analysis **S5**

D Statistical analysis **S6**

E Selection of number of centroids **S8**

F Data set selection **S8**

F.1 Chemical relatedness S9

F.2 Biological diversity S12

G Variance of performance differences decreases with increasing alignment **S12**

H Selected featurization strategy for simulating incremental data collection **S13**

References **S14**

A Diversity of the shared evaluation data set

To calculate representational alignment, we utilized a shared evaluation data set that is the union of all data sets. To assess the diversity of this data set, we summarize each property distribution by its central 95 percent range, from the 2.5th to the 97.5th percentile. The properties considered are hydrogen-bond acceptor count, hydrogen-bond donor count, molecular weight, topological polar surface area (TPSA), rotatable bond count, and logP. The corresponding values are reported in Table S1, and the wide ranges indicate substantial diversity across the data set.

Table S1: Central 95 percent ranges for molecular property distributions in the shared data set.

**Property**	**2.5th percentile**	**97.5th percentile**
# Hydrogen-bond acceptors	1	11
# Hydrogen-bond donors	0	5
Molecular weight [Da]	123.16	632.75
TPSA [Å^2]	6.48	181.62
# Rotatable bonds	0	12
logP	-0.94	6.49

B Proof of Proposition 1

Lemma B.1. *Let* $A = X(X^TX)^{-\frac{1}{2}}$ *and* $B = Z(Z^TZ)^{-\frac{1}{2}}$, *then*

$$||A^TB||_F^2 \geq \frac{||X^TZ||_F^2}{||X^TX||_F||Z^TZ||_F} \tag{1}$$

Proof.

$$
\begin{aligned}
||X^T Z||_F =& ||(X^T X)^{\frac{1}{2}}(X^T X)^{-\frac{1}{2}} X^T Z (Z^T Z)^{-\frac{1}{2}} (Z^T Z)^{\frac{1}{2}}||_F \\
\leq & ||(X^T X)^{\frac{1}{2}}||_2 ||(X^T X)^{-\frac{1}{2}} X^T Z (Z^T Z)^{-\frac{1}{2}}||_F ||(Z^T Z)^{\frac{1}{2}}||_2
\end{aligned}
$$

$\Rightarrow$

$$
\begin{aligned}
||A^T B||_F^2 =& ||(X^T X)^{-\frac{1}{2}} X^T Z (Z^T Z)^{-\frac{1}{2}}||_F^2 \\
\geq & \frac{||X^T Z||_F^2}{||(X^T X)^{\frac{1}{2}}||_2^2 ||(Z^T Z)^{\frac{1}{2}}||_2^2} \\
\geq & \frac{||X^T Z||_F^2}{||X^T X||_F ||Z^T Z||_F}
\end{aligned}
$$

□

Proof. Projection matrices P_Z are of the form $P_Z = Z(Z^T Z)^{-1} Z^T$ and $\operatorname{Tr} P_Z = \operatorname{rank} Z$.

Note that $P_X = AA^T$ and $P_Z = BB^T$. Hence

$$
\begin{aligned}
\operatorname{Tr}(P_X P_Z) &= \operatorname{Tr}(AA^T BB^T) \\
&= \operatorname{Tr}(A^T BB^T A) \\
&= \operatorname{Tr}(A^T B (A^T B)^T) \\
&= ||A^T B||_F^2
\end{aligned}
$$

$$
\begin{aligned}
||XW - y||_2 - ||ZV - y||_2 =& ||XW - ZV + ZV - y||_2 - ||ZV - y||_2 \\
\leq& ||XW - ZV||_2 + ||ZV - y||_2 - ||ZV - y||_2 \\
=& ||X(X^T X)^{-1} X^T y - Z(Z^T Z)^{-1} Z^T y||_2 \\
=& ||(P_X - P_Z)y||_2 \\
\leq& ||(P_X - P_Z)||_2 \\
\leq& ||(P_X - P_Z)||_F \\
=& \sqrt{\operatorname{Tr}((P_X - P_Z)^T (P_X - P_Z))} \\
=& \sqrt{\operatorname{Tr}(P_X^T P_X) + \operatorname{Tr}(P_Z^T P_Z) - \operatorname{Tr}(P_X^T P_Z) - \operatorname{Tr}(P_Z^T P_X)} \\
=& \sqrt{\operatorname{Tr}(P_X) + \operatorname{Tr}(P_Z) - 2 \cdot \operatorname{Tr}(P_X^T P_Z)} \\
=& \sqrt{\operatorname{rank} X + \operatorname{rank} Z - 2||A^T B||_F^2} \\
\leq& \sqrt{\operatorname{rank} X + \operatorname{rank} Z - 2 \cdot \mathrm{CKA}_{\mathrm{linear}}(X, Z)}
\end{aligned}
$$

□

C Non-aggregated APD analysis

Figure S1 is the non-aggregated equivalent of Figure 1 (a) in the main text. The figure reports on the APD analysis of 661 data sets; i.e., it illustrates 3966 model pairs, each data point showing the alignment between the two models and their performance difference. In the main text, we describe how we performed a cluster-based permutation test on per-data-set averages (Section D) and found that the sparsity in the high-alignment/high-performance-difference region is unlikely to be due to chance. Note that the same test cannot be applied to the non-aggregated data (Figure S1), as model pairs from the same data set are dependent due to each model appearing in multiple model pairs. Nonetheless, alignment still shapes

performance differences, as performance differences are above the 1 minus alignment line for only 0.4% of model pairs.

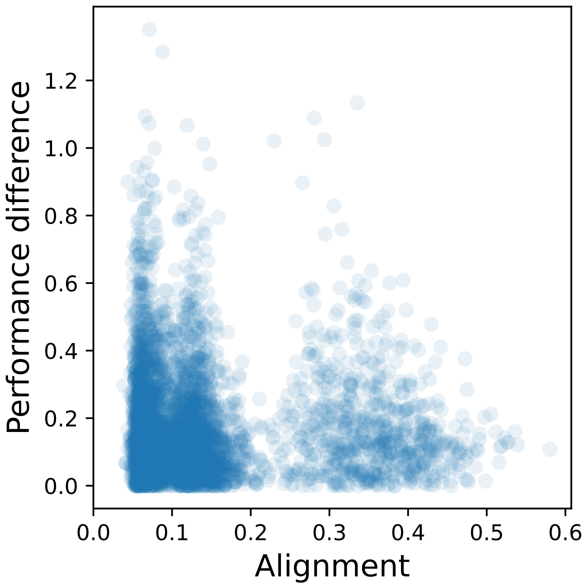

Figure S1: Comparison of performance difference and alignment for 661 data sets. Alignment of models based on PC, FP, ChemGPT, and GINC, plotted against corresponding performance differences (measured by MCC)

D Statistical analysis

To identify an exclusion zone, we require a statistical method capable of detecting unusually large regions with significantly low data density. Finding an underrepresented area in a two-dimensional space without knowing its exact location means testing every possible threshold combination, which in turn requires correcting for multiple comparisons. Given the prior knowledge that effects are likely to occur within a contiguous region, applying a strict Bonferroni correction would substantially increase the risk of Type II errors, failing to detect significant effects.

Interestingly, a similar challenge arises in neuroimaging, where researchers aim to identify brain regions that show significant differences in activity between different conditions. In

this context, measurements are collected simultaneously from multiple sensors across participants' brains to detect significant differences in signals across numerous sensor–time pairs. Addressing this issue with simple correction methods can be detrimental to the analysis, which is why the field has adopted non-parametric, cluster-based permutation tests.[1] Such tests operate sequentially by considering each (sensor, time) pair, computing a test statistic of interest, and finally applying a threshold to identify whether the observed effect is significant (e.g., $p < 0.05$). Neighboring pairs that pass this threshold are grouped into clusters, a cluster-based statistic (e.g., the sum of p-values) is computed, and the maximal cluster statistic is stored. After this is completed, the sensor values are permuted, and the previously described process is rerun to compute the maximal cluster statistic of the permuted data. This procedure is repeated multiple times, yielding a distribution of test statistics that allows inference on the significance of entire spatiotemporal patterns rather than individual comparisons.[1]

Inspired by the similarity of the underlying challenge, our analysis follows an analogous approach. In our case, each coordinate pair consists of an alignment threshold as the first value and a performance difference threshold as the second. Each threshold pair was derived from two selected percentiles of the data. The thresholds were then used to filter the model pairs that were found to exceed both thresholds. Because the thresholds were selected based on data percentiles, the number of data sets that should remain after filtering (under the assumption that no relationship exists between alignment and performance differences at these thresholds) was known. Hence, a binomial test could be employed to assess significance. P-values were computed from the binomial test statistic, and all threshold pairs with values below 0.05 were identified. Among the pairs that passed this filtering, all connected components were then identified, and the largest component was retained, for which an aggregate test statistic (the size) was computed. This analysis was repeated 10,000 times, each time after the alignment on the different data sets had been randomly shuffled and the largest cluster size had been recorded. Through this process, a distribution of the largest

cluster statistics was obtained, which can be used to form the null hypothesis that no large threshold regions exist where data sets are scarce.

E Selection of number of centroids

To determine the optimal number of clusters for evaluating whether the mean minimum Tanimoto distance predicts the approximate location in the performance difference alignment plot, we assess clustering quality using the Silhouette score, with two to six centroids tested in k-means. The results are shown in Figure S2.

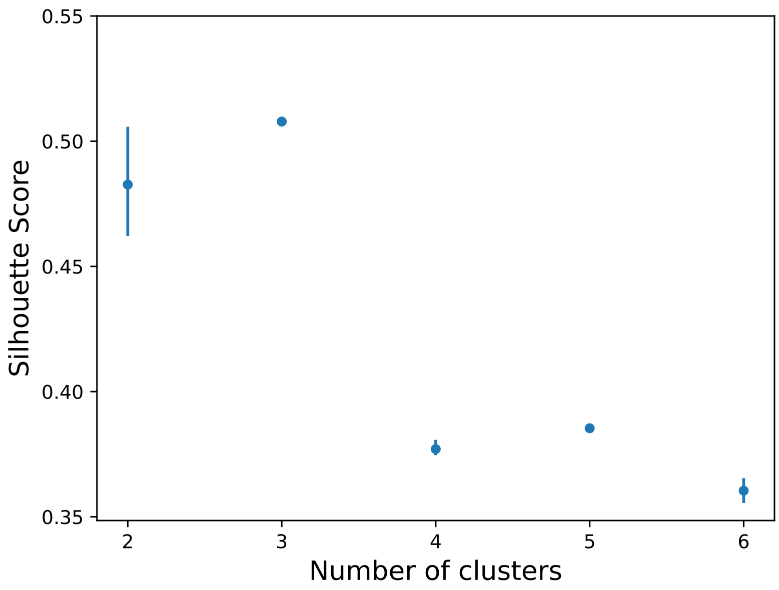

Figure S2: Average Silhouette score of varying number of clusters for the result of k-means based on 495 training data sets across 100 random initializations.

F Data set selection

To assess whether our selection of 10 data sets is representative of the larger collection (661 data sets) for TDC data sets, we compare the two data set collections based on chemical

similarity and biological diversity. The selected data sets are listed in Table S2.

Table S2: Selected data sets used in this study for more comprehensive APD analysis in terms of descriptor selection.

Data set name	Data set ID	Data set	Description	Source
5-HT1	NVS_GPCR_r5HT1_NonSelective	GPCR	Serotonin receptor binding	2,3
HepG2	APR_HepG2_StressKinase_1h_up	Cellular Pathway	HepG2 stress kinase activation	2,3
TF	ATG_Oct_MLP_CIS_dn	Transcription Factor	OCT motif activity	2,3
PTPN11	NVS_ENZ_hPTPN11	Enzyme	Tyrosine phosphatase inhibition	2,3
SULT2A	CLD_SULT2A_24hr	Metabolism	Sulfotransferase 2A gene expression	2,3
CYP1A2	CYP1A2_Veith	ADME	CYP1A2 inhibition	4
CYP3A4	CYP3A4_Veith	ADME	CYP3A4 inhibition	4
CYP2D6	CYP2D6_Veith	ADME	CYP2D6 inhibition	4
hERG	hERG_inhib	Ion Channel	hERG inhibition	5
STK	serine_threonine_kinase_33_butkiewicz	Kinase	Serine/threonine kinase inhibition	6

F.1 Chemical relatedness

Chemical relatedness between the two data set collections, specifically the full set obtained by combining all 661 data sets into a single data set and the subset of 10 selected data sets, was assessed on the basis of structural and molecular-property similarity. Structural relatedness was quantified using the minimal Tanimoto distance from one data set to the other, and property relatedness was evaluated using the number of hydrogen acceptors, molecular weight, number of hydrogen donors, TPSA, number of rotatable bonds, and LogP. All calculations were carried out using RDKit and FP2Sim.

Among the 10 selected data sets, exact matches comprised 36% of all molecules in the full set of 661 data sets. Figure S3 shows the cumulative distribution of minimum Tanimoto distances between molecules in the two collections. Over 50% of molecules in the larger collection had a nearest neighbor in the 10 selected data sets with a Tanimoto distance below 0.44.

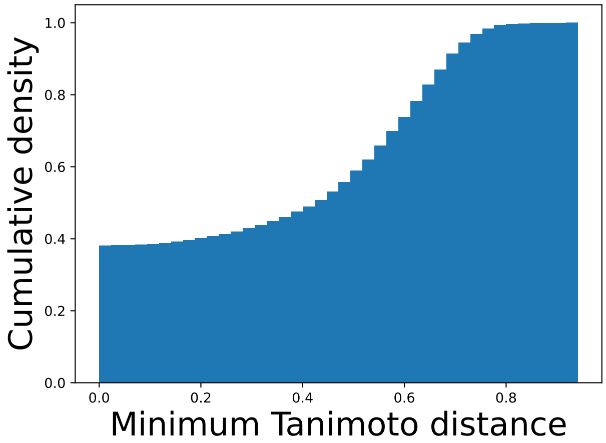

Figure S3: Cumulative density of minimum Tanimoto distance between full data set collection and 10 data sets selected for more in depth experiments.

Among the six molecular properties compared, histograms computed with 40 bins show an overlap of at least 86.1% between the full and selected data sets. Figure S4 displays these histograms along with their corresponding overlap values. Note that the overlap was calculated using aligned bins, whereas the plotted histograms use unaligned bins for improved visual presentation.

Given that the 10 data sets contain 36% exact matches and that over 50% of molecules have close structural neighbors, combined with the high overlap in property distributions, we conclude that the 10 data sets are representative in terms of chemical similarity.

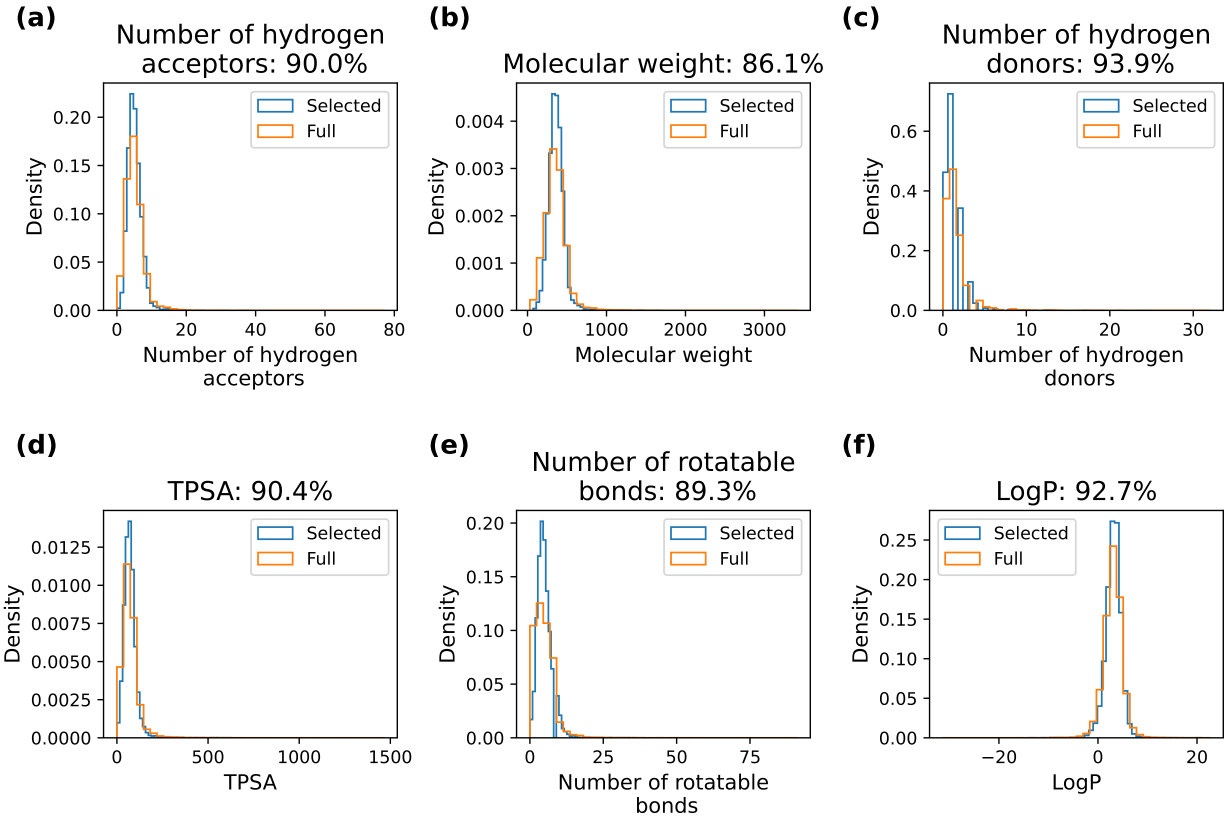

Figure S4: Histogram of 6 molecular properties for full data set collection and selected data set with calculated overlap in %.

F.2 Biological diversity

The 10 selected data sets were chosen to provide broad biological coverage while focusing on mechanistically relevant targets and pathways. They include GPCRs (e.g., NVS_GPCR_r5HT1_NonSelective_ToxCast), ion channels (e.g., hERG_inhib_herg_central), and enzymes (e.g., NVS_ENZ_hPTPN11_ToxCast), capturing major protein families of pharmacological interest. Key cellular pathways such as kinase signaling and transcription factor regulation are represented by data sets like serine_threonine_kinase_33_butkiewicz_hts and ATG_Oct_MLP_CIS_dn_ToxCast. ADME-related processes are covered through cytochrome P450 data sets (CYP1A2_Veith_adme, CYP3A4_Veith_adme, CYP2D6_Veith_adme) and other metabolic markers (CLD_SULT2A_24hr_ToxCast), while toxicity endpoints, including cardiotoxicity and stress responses, are represented by hERG_inhib_herg_central and APR_HepG2_StressKinase_1h_up_ToxCast. Collectively, these data sets ensure a representative selection across target classes, pathways, and safety-relevant effects, making them suitable for downstream analyses of biological space.

G Variance of performance differences decreases with increasing alignment

To assess how performance differences vary with alignment in our experiments with 23 featurization strategies and 10 data sets, we split all observed model pairs into 5 equally sized bins with respect to their alignment. For each bin, we calculated the variance of performance differences for each bin using bootstrapping with 1000 redraws to obtain 95% confidence intervals (Figure S5).

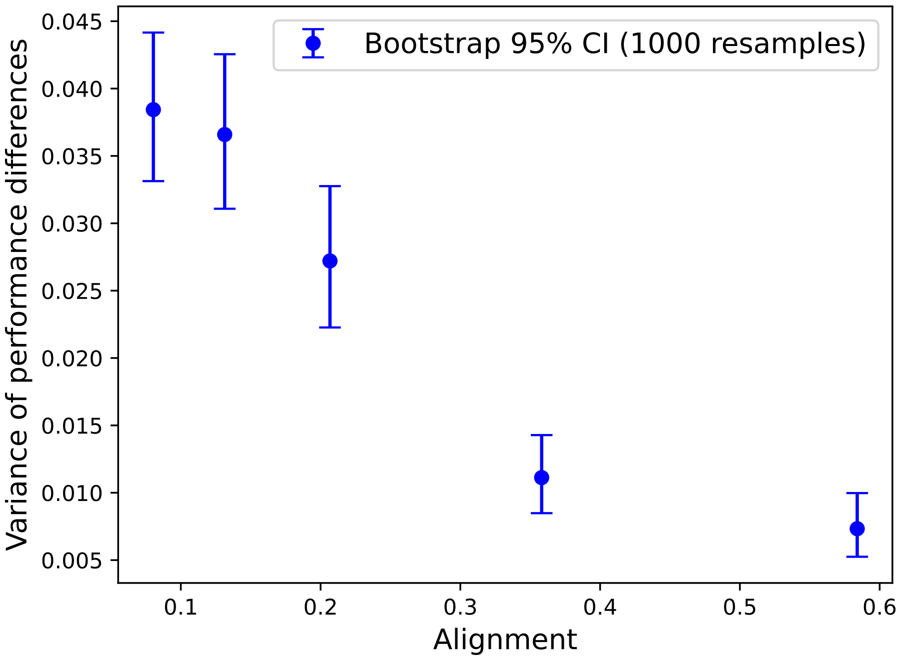

Figure S5: Performance difference variability dropped with increasing alignment.

H Selected featurization strategy for simulating incremental data collection

We randomly select one out of 23 representations to simulate a model-building scenario in which data is collected incrementally. We report which featurization strategy was chosen for which data set in Table S3. Data set names correspond to the data set names used in Table S2.

Table S3: Data sets and corresponding featurization strategies.

Data set name	Featurization strategy	layer	Length of features
5-HT1	FP	-	2048
HepG2	ChemGPT-19M	24	256
TF	gin_supervised_infomax	4	300
PTPN11	ChemGPT-4.7M	22	128
SULT2A	gin_supervised_infomax	5	300
CYP1A2	gin_supervised_infomax	5	300
CYP3A4	PC	-	215
CYP2D6	ChemGPT-19M	23	256
hERG	gin_supervised_edgepred	3	300
STK	gin_supervised_contextpred	3	300

References

(1) Maris, E.; Oostenveld, R. Nonparametric statistical testing of EEG- and MEG-data. *J. Neurosci. Methods* **2007**, *164*, 177–190.

(2) Feshuk, M. Assay description documents. `https://clowder.edap-cluster.com/datasets/6894f2dae4b025654d12b716?space=687e388ce4b02565bc3e28e4#`, 2025; Accessed: 21. Nov 2025.

(3) Richard, A. M. et al. ToxCast chemical landscape: Paving the road to 21st century toxicology. *Chem. Res. Toxicol.* **2016**, *29*, 1225–1251.

(4) Veith, H.; Southall, N.; Huang, R.; James, T.; Fayne, D.; Artemenko, N.; Shen, M.; Inglese, J.; Austin, C. P.; Lloyd, D. G.; Auld, D. S. Comprehensive characterization of cytochrome P450 isozyme selectivity across chemical libraries. *Nat. Biotechnol.* **2009**, *27*, 1050–1055.

(5) Du, F.; Yu, H.; Zou, B.; Babcock, J.; Long, S.; Li, M. hERGCentral: A large database to store, retrieve, and analyze compound-human ether-à-go-go related gene channel interactions to facilitate cardiotoxicity assessment in drug development. *Assay Drug Dev. Technol.* **2011**, *9*, 580–588.

(6) Butkiewicz, M.; Lowe, E.; Mueller, R.; Mendenhall, J.; Teixeira, P.; Weaver, C.; Meiler, J. Benchmarking ligand-based virtual high-throughput screening with the PubChem database. *Molecules* **2013**, *18*, 735–756.
